# Supplementary material for: Developing an Updated Strategy for Estimating the Free-Energy Parameters in RNA Duplexes
Source: Int J Mol Sci. 2021 Sep 8;22(18):9708. doi: 10.3390/ijms22189708 (PMC8467000; doi:10.3390/ijms22189708)
Supplement: Supplementary file 1 [file ijms-22-09708-s001.zip › ijms-1343883-supplementary.pdf]

Developing an updated strategy for estimating the free energy parameters in RNA duplexes

Wayne Dawson [1,]*, Amiu Shino [1], Gota Kawai [2], and Ella Czarina Morishita [1,]*

[1] Veritas In Silico, 1-11-1 Nishigotanda, Shinagawa-ku, Tokyo 141-0031; wkd@veritasinsilico.com (W.D.); ars@vi14si.com (A.S.); ecm@vi14si.com (E.C.M.)
[2] Department of Life Science, Faculty of Advanced Engineering, Chiba Institute of Technology, 2-17-1 Tsudanuma, Narashino-shi, Chiba 275-0016, Japan; gota.kawai@p.chibakoudai.jp
* Correspondence: wkd@veritasinsilico.com (W.D.); ecm@vi14si.com (E.C.M.)

Special Issue: RNA Informatics

Website: https://www.mdpi.com/journal/ijms/special_issues/RNA_Informatics

Guest Editor: Prof. Dr. Michiaki Hamada & Prof. Dr. Akito Taneda

Methods Supplement:

Relation between absorption and concentration

The *absorption* ($A(T)$) measures the concentration of unfolded or single-stranded RNA (ssRNA). From Beer's law, the temperature dependence of absorption is defined as:

$$A(T) = Elc_{s\text{-}s}(T) \tag{S1}$$

where E is the extinction coefficient, l is the optical path length from the light source, and $c_{s\text{-}s}(T)$ is the concentration of unfolded RNA (i.e., ssRNA) as a function of temperature. This can be written in terms of the mole fraction of ssRNA as follows:

$$A(T) = Elc_T\alpha\ , \tag{S2}$$

where α is the temperature-dependent mole fraction of ssRNA and c_T is a constant that will be defined later.

How do we define α? Suppose we have two non-self-complementary strands (A) and (B) forming a duplex (AB). The mole fraction of sequence A is χ_{A} and of sequence B is χ_{B}. Let

$$\alpha = 2\min\{\chi_{\mathrm{A}}, \chi_{\mathrm{B}}\}\ ; \tag{S3}$$

i.e., twice the mole fraction of the sequence with the smallest concentration of ssRNA. This condition is selected because it need not be the case that $\chi_{\mathrm{A}} = \chi_{\mathrm{B}}$; however, the maximum amount of double-stranded RNA (dsRNA) that can form is limited to $\min\{\chi_{\mathrm{A}}, \chi_{\mathrm{B}}\}$ and twice this minimum is the maximum amount of ssRNA that can form as a result of melting the dsRNA duplex. Any excess of A or B is irrelevant.

The mass balance equation and mole fractions for this non-self-complementary RNA duplex system is derived as follows:

$$\begin{array}{ccccc} \mathrm{A} & + & \mathrm{B} & \rightleftharpoons & \mathrm{AB} \\ c_T\alpha/2 & & c_T\alpha/2 & & c_T(1-\alpha)/2 \end{array}$$

where based on Equation (S3), the mole fractions are:

$$\chi_{\mathrm{A}} = \alpha/2\ ,\ \chi_{\mathrm{B}} = \alpha/2 \text{ and } \chi_{\mathrm{AB}} = (1-\alpha)/2\ . \tag{S4}$$

From Equations (S3) and (S4), we can write the concentrations as

$$[\mathrm{A}] = c_T\alpha/2\ ,\ [\mathrm{B}] = c_T\alpha/2 \text{ and } [\mathrm{AB}] = c_T(1-\alpha)/2\ , \tag{S5}$$

and c_T is defined as

$$c_T = 2\min\{[\mathrm{A}]_{init}, [\mathrm{B}]_{init}\}, \tag{S6}$$

where $[\mathrm{A}]_{init}$ and $[\mathrm{B}]_{init}$ are the initial concentrations of sequence A and B, respectively. Why does the factor of "2" appear in Equation (S6), because it is related to α, which was defined in Equation (S3) to be the sum of the *minimum* (the smallest) mole fraction, i.e., $\alpha = 2\min\{\chi_\mathrm{A}, \chi_\mathrm{B}\}$. Note that the form of Equation (S6) only applies when sequences A and B are not the same. If we are working with *self-complementary* sequences, then $c_T = [\mathrm{A}]_{init}$. Although c_T is a convenient shorthand, its definition is unfortunately context dependent and evolved *somewhat* haphazardly in the course of history.

The concentration dependent equilibrium constant for *non-self-complementary* sequences becomes

$$K_n(\alpha) = \frac{[\mathrm{AB}]}{[\mathrm{A}][\mathrm{B}]} \rightarrow \frac{\left(c_T(1-\alpha)/2\right)}{[c_T\alpha/2]^2} = \frac{2(1-\alpha)}{c_T\alpha^2}. \tag{S7}$$

In the case of *self-complementary* RNA strands, half the duplex ends up as one strand, and half the other. Since there is only one concentration [A], Equation (S3) becomes

$$\alpha = \chi_\mathrm{A}, \tag{S8}$$

and likewise, Equation (S4) becomes

$$\chi_\mathrm{A} = \alpha \text{ and } \chi_{\mathrm{A}_2} = (1-\alpha)/2, \tag{S9}$$

and Equation (S5) becomes

$$[\mathrm{A}] = c_T\alpha \text{ and } [\mathrm{A}_2] = c_T(1-\alpha)/2. \tag{S10}$$

Therefore, the chemical mass balance equation becomes

$$\begin{array}{ccc} 2\mathrm{A} & \rightleftharpoons & \mathrm{A}_2 \\ c_T\alpha & & c_T(1-\alpha)/2 \end{array},$$

and the concentration dependent equilibrium constant (for the self-complementary case) is

$$K_s(\alpha) = \frac{[\mathrm{A}_2]}{[\mathrm{A}]^2} \rightarrow \frac{\left(c_T(1-\alpha)/2\right)}{[\alpha c_T]^2} = \frac{(1-\alpha)}{2c_T\alpha^2}. \tag{S11}$$

We define η to be 1 for self-complementary duplexes and 4 for non-self-complementary duplexes

$$\eta = \begin{cases} 1, & \text{self-complementary duplex} \\ 4, & \text{non-self-complementary duplex} \end{cases}. \tag{S12}$$

This permits us to simplify Equations (S7) and (S11) to the following:

$$K_{eq} = \frac{\eta(1-\alpha)}{2c_T\alpha^2}\,, \tag{S13}$$

where K_{eq} is the concentration dependent equilibrium constant corresponding to Equations (S7) and (S11). At the melting temperature T_m, we have $\alpha = 1/2$, Equation (S11) becomes $K_s(\alpha = 1/2) = 1/c_T$ and Equation (S7) becomes $K_n(\alpha = 1/2) = 4/c_T$. Hence, at T_m,

$$K_{\alpha=1/2} = \eta / c_T\,. \tag{S14}$$

Relation between thermodynamics and equilibrium

The thermodynamics of RNA melting are expressed as

$$\Delta G = \Delta H - T\Delta S\,, \tag{S15}$$

where ΔG is the free energy, ΔH is the enthalpy, and ΔS is the entropy. In terms of chemical equilibrium, this is expressed as

$$K_{eq} = \exp\left(-\frac{\Delta G}{k_B T}\right) = \frac{\eta(1-\alpha)}{2c_T\alpha^2}\,, \tag{S16}$$

where k_B is Boltzmann constant; In the units used here, $k_B = R \sim 0.001987$ kcal/mol. Then,

$$\Delta G = -k_B T \ln\left(\frac{\eta(1-\alpha)}{2c_T\alpha^2}\right) = \Delta H - T\Delta S\,. \tag{S17}$$

By rearranging, we get

$$\frac{1}{T} = -\frac{k_B}{\Delta H}\ln\left(\frac{\eta(1-\alpha)}{2c_T\alpha^2}\right) + \frac{\Delta S}{\Delta H}\,. \tag{S18}$$

Now we try writing this expression at T_m, where $\alpha = 1/2$ or $K_{1/2} = \eta / c_T$, then

$$\frac{1}{T_m} = -\frac{k_B \ln(\eta / c_T)}{\Delta H^o} + \frac{\Delta S^o}{\Delta H^o}\,. \tag{S19}$$

How the Turner energy rules are determined

The Turner rules are largely based on measuring T_m in terms of different values of c_T and assuming that $\Delta G = \Delta H^o - T\Delta S^o$ is a linear function, where ΔH^o and ΔS^o are constants. This means that when plotting $1/T_m$ with respect to $\ln(\eta / c_T)$ for different values of c_T, such as

$$\frac{1}{T_{m,1}} = -\frac{k_{\mathrm{B}} \ln(\eta / c_{T,1})}{\Delta H^o} + \frac{\Delta S^o}{\Delta H^o} \text{ and } \frac{1}{T_{m,2}} = -\frac{k_{\mathrm{B}} \ln(\eta / c_{T,2})}{\Delta H^o} + \frac{\Delta S^o}{\Delta H^o}, \quad \text{(S20a)}$$

we have two equations and two unknowns. Turner used linear regression in fitting Equation (S20a) to obtain parameters for ΔS^o and ΔH^o [1]. This means that the results are not based upon a fit of the melting curves; rather, it is a fit of the measurements of T_m for at least two concentrations. Using the solution for ΔS^o and ΔH^o for melting of the whole sequence, these parameters are further broken down into sub-parameters and solved by

$$\Delta G^o = \sum_{NN} \Delta G^o_{NN}, \ \Delta H^o = \sum_{NN} \Delta H^o_{NN}, \text{ and } \Delta S^o = \sum_{NN} \Delta S^o_{NN}, \quad \text{(S20b)}$$

where for the individual nearest neighbor (NN) base pairs parameters are expressed as ΔG^o_{NN}, ΔH^o_{NN} and ΔS^o_{NN} [1] for free energy, enthalpy and entropy, respectively. This approach helps circumvents the need to determine α because $T_{m,1}$.is dependent on $c_{T,1}$ and $T_{m,2}$ solely on $c_{T,2}$.

How to determine α from the experimental T_m measurements

Another way to solve for the free energy of the sequence is to work directly from fitting the equation $A(T)$ and solving this relation such that $\alpha = \alpha_{s\text{-}s}$, where $\alpha_{s\text{-}s}$ is the single-stranded fraction, and $\alpha_{d\text{-}s}$ is the double-stranded fraction (our actual goal).

Direct approach from the fit

In Equation (S1), we expressed an idealized $A(T)$ where the initial concentration of single-stranded RNA, $c_{s\text{-}s}(T)$ must be 0 for $T \ll T_m$ and c_T for $T \gg T_m$, where c_T is the maximum possible concentration of single-stranded RNA after the RNA duplex is melted. Note that c_T must be twice the quantity of dsRNA; half of c_T comes from the one sequence and half from the other.

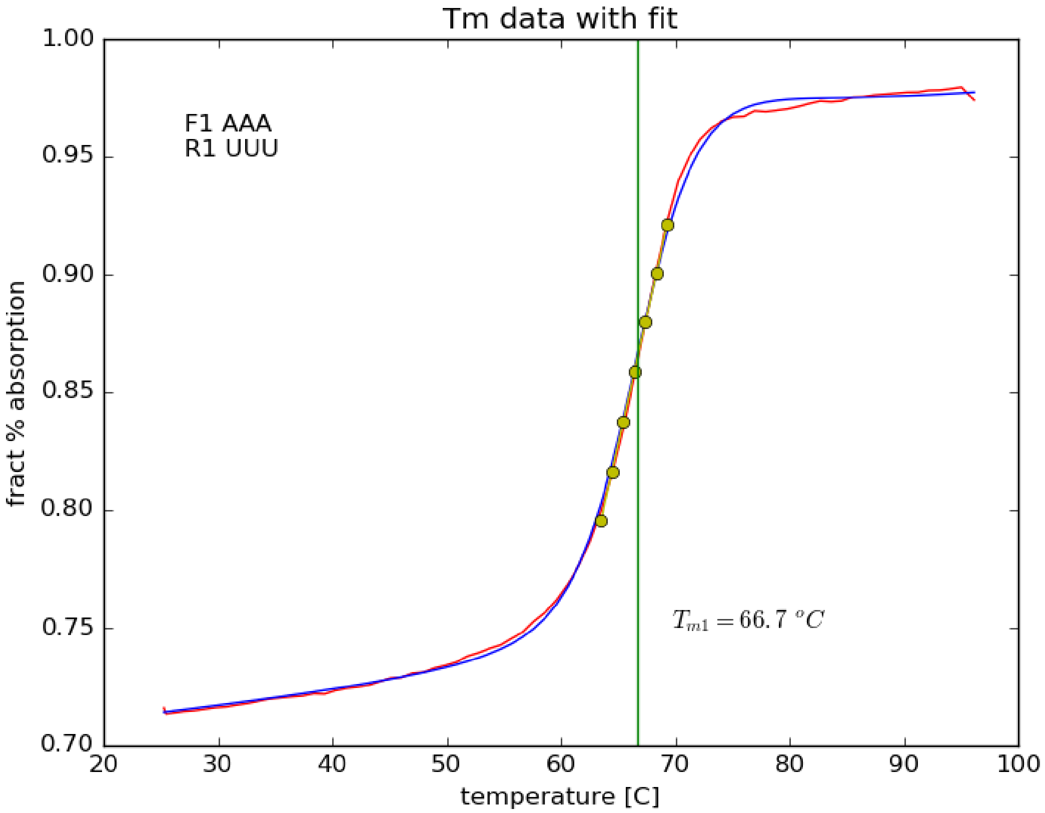

Figure S1. Fit of the experimental data including a linear fit of the region around T_m.

Although this is the theory according to Beer's law, this is not exactly how **Figure S1** comes out. For example, a baseline background signal (A_{bg0}) around 0.7 is quite visible in **Figure S1** and so the maximum value for $A(T)$ is not A_{mx} but some combination of several contributions. A more accurate approximation of the data in **Figure S1** is that of the following function:

$$\bar{A}(T) = A_{bg0} + \frac{A_{mx}}{1 + \exp[-a(T - T_m)]} + A_{bg1}T \quad \text{(S21)}$$

where A_{bg0} is the absorption background baseline, A_{mx} is the absorption maximum, A_{bg1} expresses a linear temperature dependent background contribution, and a expresses the spread of the sigmoid function (roughly $\pm 5°\ \mathrm{C}$) in **Figure S1**.

Note, in reality, the fit does not only require corrections like A_{bg0} and A_{bg1}, but there is at least one additional sigmoid temperature component. Moreover, it is not entirely clear what precisely causes

the values for A_{bg0} and A_{bg1}; most likely other absorbing materials in the sample, such as water, the glass cuvette that holds the sample, and possibly some excess ssRNA, all contribute some part of A_{bg0} and the differential measuring method contains a net-positive-increasing linear component (A_{bg1}).

All we are interested in within this measurement is the net relative change of single-stranded RNA. Hence, we want Equation (S1) to be expressed in the following form:

$$A(T) = Elc_{s\text{-}s}(T) = \frac{Elc_T}{1+\exp[-a(T-T_m)]} \qquad \text{(S22)}$$

where it can be seen that $A(T \ll T_m) = 0$ and $A(T \gg T_m) = Elc_T$. Equation (S22) permits us to work backwards and find the sigmoid function

$$Elc_{s\text{-}s}(T) = \left(\overline{A}(T) - A_{bg0} - A_{bg1}T\right) = \frac{A_{mx}}{1+\exp[-a(T-T_m)]} \qquad \text{(S23)}$$

Hence, $A_{mx} = 2Elc_T$. It follows that

$$c_{s\text{-}s}(T) = \frac{\overline{A}(T) - A_{bg0} - A_{bg1}T}{El} = \frac{c_T}{1+\exp[-a(T-T_m)]} \qquad \text{(S24)}$$

Equation (S24) can be easily rewritten in terms of $\alpha = \alpha_{s\text{-}s}$

$$c_{s\text{-}s}(T) = c_T\alpha_{s\text{-}s} = \frac{c_T}{1+\exp[-a(T-T_m)]} \qquad \text{(S25)}$$

We see that $c_{s\text{-}s}(T \ll T_m) \approx 0$ and $c_{s\text{-}s}(T \gg T_m) \approx c_T$.

Since our focus is not on the amount of ssRNA but on the amount of duplex, we need to write the expressions in terms of $\alpha_{d\text{-}s}$. How do we do that? Let us work from the concept of concentration. We know from Equation (S25) that $c_{s\text{-}s} = c_T\alpha_{s\text{-}s}$. From Equation (S3), we know that $\alpha_{s\text{-}s} = 2\min\{\chi_{\mathrm{A}}, \chi_{\mathrm{B}}\}$ for the non-complementary sequence duplex and from Equation (S9) that $\alpha_{s\text{-}s} = \chi_{\mathrm{A}}$ for the complementary sequence duplex. We can also deduce that $c_{d\text{-}s} = c_T\alpha_{d\text{-}s}$, since (particularly for the non-complementary case) it is easy to see that each ssRNA concentration is c_T and each ssRNA sequence can only contribute a quantity c_T to the total concentration of dsRNA. Therefore, we reason as follows:

Let $c_{s\text{-}s} = c_T\alpha_{s\text{-}s}$ and $c_{d\text{-}s} = c_T\alpha_{d\text{-}s}$.
Then $c_{d\text{-}s} = 0.5(1-\alpha_{s\text{-}s})c_T$ and it follows from the definitions that
$c_{d\text{-}s} = c_T(1-\alpha_{s\text{-}s})/2 \Rightarrow (c_T/2)\alpha_{d\text{-}s} = c_T(1-\alpha_{s\text{-}s})/2$

Hence,

$$\alpha_{d\text{-}s} = (1-\alpha_{s\text{-}s}) \text{ or } \alpha_{s\text{-}s} = (1-\alpha_{d\text{-}s}) \tag{S26}$$

Using the fact that $\alpha_{d\text{-}s} = (1-\alpha_{s\text{-}s})$, we solve Equation (S25) for $\alpha_{s\text{-}s}$ and use Equation (S26) to obtain

$$\alpha_{d\text{-}s} = 1 - \left[\frac{1}{1+\exp[-a(T-T_m)]}\right] = 1 - \left[\frac{\bar{A}(T) - A_{bg0} - A_{bg1}T}{A_{mx}}\right] \tag{S27}$$

where $\bar{A}(T)$ and T are the experimentally obtained data and A_{bg0}, A_{bg1} and A_{mx} are obtained by fitting using Equation (S21). Now we have $\alpha_{d\text{-}s}$ and the T-dependence of the experimental data. Equation (S27) is shown in **Figure S2**.

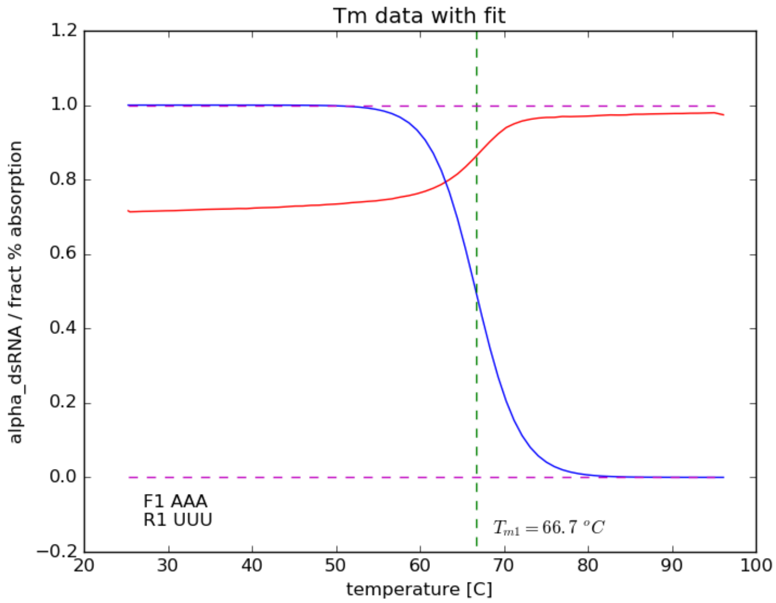

Figure S2. Result of fitting the melting data to as a function of the concentration of dsRNA using Equation S27.

Obtaining the linear fit in Figure S1 around T_m

Now that we have developed the formal theory, we can approach the problem a little more directly with the information above; we just fit the linear part of the curve around T_m with the help of Equation (S27).

Figure S1 shows a linear fit of the curve around T_m; a narrow range plus and minus 3 degrees around T_{m1} in the figure. In this narrow range, the experimental absorption data can be approximated as

$$\bar{A}(T) = \left(\frac{\Delta A}{\Delta T}\right)(T - T_m) + A_{1/2}, \tag{S28}$$

where $A_{1/2}$ is the absorption when there is only half the total concentration of dsRNA, and $\Delta A / \Delta T$ is assumed to be a constant in the region around $A_{1/2}$ where the increase in absorption is approximately linear. Based on fitting the curve in **Figure S1** to a sigmoidal function we can obtain

$$A(T) = Elc_{s\text{-}s}(T) = 2(1 - \alpha_{d\text{-}s})c_T El = \left[\left(\frac{\Delta A}{\Delta T}\right)(T - T_m) + A_{1/2}\right] - A_{bg0} - A_{bg1}T \tag{S29}$$

or, rearranging the expression into linear components and using the fact that $A_{mx} = 2c_T El$

$$\alpha_{d\text{-}s} = 1 - \left[\left(\Delta A / \Delta T - A_{bg1}\right)T + \left(A_{1/2} - \left(\Delta A / \Delta T\right)T_m - A_{bg0}\right)\right] / A_{mx} \tag{S30}$$

where the parameters A_{bg0}, A_{bg1}, A_{mx} and T_m come from fitting the expression in terms of the sigmoid function in Equation (S27) and $\Delta A / \Delta T$ and $A_{1/2}$ are from Equation (S28) and are obtained by fitting the linear part of **Figure S1** around T_m.

Both Equations (S27) and (S30) yield similar results. It is absolutely essential to include the corrections from A_{bg0}, A_{bg1}, and A_{mx} in Equation (S30) or the calculations fail to make sense.

How to obtain the free energy parameters from the duplex melting data

To obtain the free energy of the duplex, we assume that ΔH^o and ΔS^o are constants and use Equations (S7) or (S11) to express $\alpha_{d\text{-}s}$ in terms of Equation (S18)

$$\frac{1}{T} = -\frac{k_B \ln\left(K_{eq}(\alpha)\right)}{\Delta H^o} + \frac{\Delta S^o}{\Delta H^o} \tag{S31}$$

where $K_{eq} = \eta\alpha_{d\text{-}s} / 2c_T(1 - \alpha_{d\text{-}s})^2$. We now plot $1/T$ vs $k_B \ln(K_{eq})$, as in **Figure S3** (red line and dots). Fitting this expression to a linear equation, we obtain a slope and intercept $m = 1/\Delta H^o$ and $b = -\Delta S^o / \Delta H^o$, respectively. The fit is shown in **Figure S3** (blue dash). This yields the free energy for the particular melting element fitted. Note that there is a small amount of curvature of this line, indicating that a quadratic term could also be fitted. Presently, we treat this as linear.

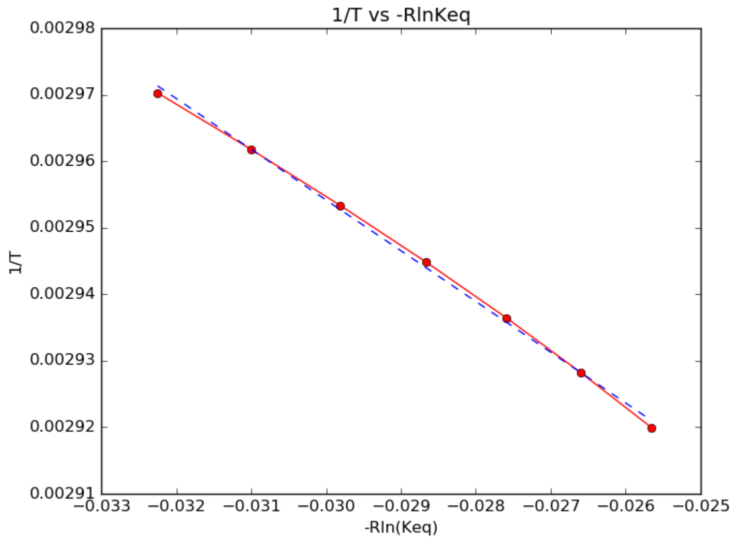

Figure S3. Fit of the inverse temperature based on the obtained concentration

As in the case of the original Turner energy rules method, based upon the solution for ΔS^o and ΔH^o of the whole sequence, parameters for the individual base pairs could be obtained from fitting for the dinucleotide base pairs, ΔS^o_{NN} and ΔH^o_{NN} in Equation (S20b) from the free energy in Equation (S20a). The basic procedures for obtaining ΔG^o_{NN} (ΔG_{NN} at 37°C), ΔH^o_{NN} and ΔS^o_{NN} from ΔH^o, ΔS^o, and T_m are explained in works such as references [1-3].

Alternative derivation using $\alpha_{d\text{-}s}$ without first dancing around $\alpha_{s\text{-}s}$

In the previous sections, expressions for K_{eq} and c_T in terms of $\alpha_{s\text{-}s}$ were derived. However, because hypochromicity is a measure of how much dsRNA is present, why not start from the product rather than the reactants in the mass balance equation?

Again, we start with the non-self-complementary case. Instead of defining α in terms of the sum of all the concentration of ssRNA, we could also define it in terms of the fraction of dsRNA of the target duplex

$$\alpha_{d\text{-}s} = \chi_{\mathrm{AB}} \qquad \text{(S32)}$$

and we define the absorption in the following way:

$$A(T) = 2c_T(1-\alpha_{d\text{-}s})El \qquad \text{(S33)}$$

where Equation (S26) must follow, i.e., $\alpha_{s\text{-}s} = (1-\alpha_{d\text{-}s})$. We also assume $2c_T$ instead of c_T because the experimental data is a measure of the hypochromicity (and the resulting transparency) of the sample when only dsRNA is present and the loss of hypochromicity as more and more ssRNA appears. Therefore,

$$c_{d\text{-}s} = c_T \alpha_{d\text{-}s} \tag{S34}$$

Using the definitions in Equation (S6), this means that

$$c_{s\text{-}s} = 2c_T \alpha_{s\text{-}s} \mid c_T = \min\{[\mathrm{A}]_{init}, [\mathrm{B}]_{init}\} \tag{S35}$$

i.e., twice c_T yields the total concentration of usable ssRNA. As before, this condition is selected because it need not be the case that $\chi_\mathrm{A} = \chi_\mathrm{B}$ but the minimum mole fraction will be the limiting case.

The mass balance equation and mole fractions for this non-self-complementary RNA duplex system is derived as follows:

$$\begin{array}{ccccc} \mathrm{A} & + & \mathrm{B} & \rightleftharpoons & \mathrm{AB} \\ c_T(1-\alpha_{d\text{-}s}) & & c_T(1-\alpha_{d\text{-}s}) & & c_T\alpha_{d\text{-}s} \end{array}$$

and

$$K_n(\alpha_{d\text{-}s}) = \frac{[\mathrm{AB}]}{[\mathrm{A}][\mathrm{B}]} \rightarrow \frac{c_T\alpha_{d\text{-}s}}{[c_T(1-\alpha_{d\text{-}s})]^2} = \frac{\alpha_{d\text{-}s}}{c_T(1-\alpha_{d\text{-}s})^2} \tag{S36}$$

Likewise, for the self-complementary RNA strands, the equilibrium mass balance equation becomes

$$\begin{array}{ccc} 2\mathrm{A} & \rightleftharpoons & \mathrm{A}_2 \\ 2c_T(1-\alpha_{d\text{-}s}) & & c_T\alpha_{d\text{-}s} \end{array}$$

and the concentration dependent equilibrium constant (for the self-complementary case) is

$$K_s(\alpha) = \frac{[\mathrm{A}_2]}{[\mathrm{A}]^2} \rightarrow \frac{(c_T\alpha_{d\text{-}s})}{[2(1-\alpha_{d\text{-}s})c_T]^2} = \frac{\alpha_{d\text{-}s}}{4c_T(1-\alpha_{d\text{-}s})^2} \tag{S37}$$

So, in fact, we have to define η in Equation (S12) slightly differently as η':

$$\eta' = \begin{cases} 4, & \text{self-complementary duplex} \\ 1, & \text{non-self-complementary duplex} \end{cases} \tag{S38}$$

It follows that the general expression becomes

$$K_{eq} = \frac{\alpha_{d\text{-}s}}{\eta' c_T (1-\alpha_{d\text{-}s})^2} \tag{S39}$$

and

$$K_{1/2} = 1/\eta' c_T \,. \tag{S40}$$

As can be seen, Equations (S39) and (S40) look a little different from Equations (S13) and (S14). This means they are also different from the Turner definitions presented in the proposed Turner energy rules. In Equation (S16), it was $K_{eq} = \eta(1-\alpha_{s\text{-}s})/2c_T\alpha_{s\text{-}s}^2$ whereas here, it is $K_{eq} = \alpha_{d\text{-}s}/\eta' c_T (1-\alpha_{d\text{-}s})^2$.

How do we show that we arrive at something similar to Equation (S16)?

Let's go back to the definition of c_T in Equation (S6); i.e., $c_T = 2\min\{[\mathrm{A}]_{init}, [\mathrm{B}]_{init}\}$ where $[\mathrm{A}]_{init}$ and $[\mathrm{B}]_{init}$ are the initial concentrations of sequence A and B, respectively. Then

$$A(T) = c_T (1-\alpha_{d\text{-}s}) El \tag{S41}$$

using the same reasoning as in Equation (S33), and because the maximum final concentration of the duplex is $c_T/2$, we conclude that

$$c_{d\text{-}s} = c_T \alpha_{d\text{-}s}/2 \tag{S42}$$

Similarly, we can conclude that the effective concentrations of the respective ssRNAs will be

$$[\mathrm{A}] = c_T (1-\alpha_{d\text{-}s})/2 \text{ and } [\mathrm{B}] = c_T (1-\alpha_{d\text{-}s})/2 \tag{S43}$$

The mass balance equation and mole fractions for this non-self-complementary RNA duplex system is derived as follows:

$$\underset{\frac{c_T(1-\alpha_{d\text{-}s})}{2}}{\mathrm{A}} \; + \; \underset{\frac{c_T(1-\alpha_{d\text{-}s})}{2}}{\mathrm{B}} \; \rightleftharpoons \; \underset{\frac{c_T\alpha_{d\text{-}s}}{2}}{\mathrm{AB}}$$

and

$$K_n(\alpha_{d\text{-}s}) = \frac{[\mathrm{AB}]}{[\mathrm{A}][\mathrm{B}]} \rightarrow \frac{c_T\alpha_{d\text{-}s}/2}{[c_T(1-\alpha_{d\text{-}s})/2]^2} = \frac{2\alpha_{d\text{-}s}}{c_T(1-\alpha_{d\text{-}s})^2} \tag{S44}$$

Likewise, for the self-complementary RNA strands, the chemical equilibrium balance equation becomes

$$
\begin{array}{ccc}
2\text{A} & \rightleftharpoons & \text{A}_2 \\
c_T(1-\alpha_{d\text{-}s}) & & c_T\alpha_{d\text{-}s}/2
\end{array}
$$

and the concentration dependent equilibrium constant (for the self-complementary case) is

$$K_s(\alpha_{d\text{-}s}) = \frac{[\text{A}_2]}{[\text{A}]^2} \to \frac{(c_T\alpha_{d\text{-}s}/2)}{[(1-\alpha_{d\text{-}s})c_T]^2} = \frac{\alpha_{d\text{-}s}}{2c_T(1-\alpha_{d\text{-}s})^2} \quad \text{(S45)}$$

Now we can directly apply Equation (S12) as originally defined to produce

$$K_{eq} = \frac{\eta\alpha_{d\text{-}s}}{c_T(1-\alpha_{d\text{-}s})^2} \quad \text{(S46)}$$

and

$$K_{1/2} = \eta / c_T, \quad \text{(S47)}$$

which are identical to the expressions found in all the published papers by the Turner group [1], Tinoco et al. [3], Applequist et al. [4], etc.

Like it or not, the definition of c_T ends up being a bit enigmatic no matter what we do. Suffice it to say that when one works with *non*-self-complementary sequences A and B, it must read c_T to mean

$$c_T = 2\min\{[\text{A}]_{init}, [\text{B}]_{init}\} \quad \text{(S48)}$$

And when one works with *self-complementary* sequences A and B, it must read c_T to mean

$$c_T = [\text{A}]_{init}. \quad \text{(S49)}$$

In free energy calculations as in Equations (1) and (2) in the main text, this difference between non-self-complementary and self-complementary sequences is accounted for by invoking ΔG_{sym}, so the matter is absorbed as an entropy correction

$$\Delta G_{sym} = k_{\text{B}}T\ln(2) \quad \text{(S50)}$$

It actually involves another merry-go-round logic to work from the experimental equations and arrive at Equation (S50), but at least in merely doing the calculations, it is a straight-forward procedure.

Methods used to obtain RNA melting data

Design of RNA sequences to collect nearest neighbor parameters

RNA sequences for measuring the UV melting curve were designed as 17 bp duplexes and formed by mixing equal proportions of the sequence and its complement. The resulting duplexes consisted of 64 patterns of forward strands [5’-AUGAUCG$X_1X_2X_3$CGUACUG-3’] and 64 patterns of reverse strands [5’-CAGUACG$Y_3Y_2Y_1$CGAUCAU-3’], with three random base pair in the middle. In this study, only complementary conjugates pairs were included for X_1-Y_1, X_2-Y_2, and X_3-Y_3. Oligo-ribonucleotides were ordered from Hokkaido System Science Co., Ltd. (Japan).

UV melting experiments

UV absorbance measurements were used to obtain the thermodynamic parameters of RNA duplexes. The UV melting experiments were performed in V-730BIO Spectrophotometer (JASCO Corporation, Japan) using 10 mm path length quartz cuvettes. Absorbance at 260 nm was monitored and data was collected by heating to 25 to 100 °C at 1 °C / min. Solution conditions were 2.5 µM RNA duplex, 150 mM NaCl, 20 mM sodium phosphate (pH 7.0). Nearest neighbor data were collected the total of 572 sets: complete match duplex 64 sets, one mismatch in N_2 base 192 sets, one mismatch in N_3 base 192 sets, two mismatches with the same nucleotides 48 sets, three mismatches with the same nucleotides 12 sets and GU wobble pair 64 sets.

Table S1. Benchmark comparisons of the Turner energy rules [1] with the original and new free energy prediction methods for the standard benchmarks.

	original method			new method		
sequence	fitted weight (fit) [kcal/mol]	experimental Tm data (exp) [kcal/mol]	difference (exp-fit) [kcal/mol]	fitted weight (fit) [kcal/mol]	experimental Tm data (exp) [kcal/mol]	difference (exp-fit) [kcal/mol]
ccgg~ccgg	-4.254	-4.55	-0.296	-4.095	-4.55	-0.455
cgcg~cgcg	-3.435	-3.66	-0.225	-3.274	-3.66	-0.386
gcgc~gcgc	-4.700	-4.61	0.090	-4.538	-4.61	-0.072
ggcc~ggcc	-5.519	-5.37	0.149	-5.359	-5.37	-0.011
acgca~ugcgu	-5.199	-4.97	0.229	-5.178	-4.97	0.208
agcga~ucgcu	-5.327	-5.05	0.277	-5.313	-5.05	0.263
cacag~cugug	-4.255	-4.70	-0.445	-4.231	-4.70	-0.469
cacug~cagug	-4.255	-3.34	0.915	-4.231	-3.34	0.891
gcacg~cgugc	-5.965	-6.17	-0.205	-5.944	-6.17	-0.226
gcucg~cgagc	-6.093	-6.14	-0.047	-6.079	-6.14	-0.061
accggu~accggu	-8.013	-8.51	-0.497	-8.039	-8.51	-0.471
agagag~cucucu	-6.391	-6.81	-0.419	-6.414	-6.81	-0.396
agcgcu~agcgcu	-8.025	-7.99	0.035	-8.053	-7.99	0.063
aggccu~aggccu	-8.844	-8.36	0.484	-8.874	-8.36	0.514
augcau~augcau	-4.467	-4.73	-0.263	-4.511	-4.73	-0.219
cacgug~cacgug	-6.290	-6.59	-0.300	-6.307	-6.59	-0.283
cagcug~cagcug	-7.121	-6.68	0.441	-7.142	-6.68	0.462
caugcg~cgcaug	-6.890	-7.00	-0.11	-6.914	-7.00	-0.086
ccaugg~ccaugg	-7.239	-7.30	-0.061	-7.273	-7.30	-0.027
ccgcgg~ccgcgg	-10.095	-9.84	0.255	-10.125	-9.84	0.285
ccuagg~ccuagg	-7.418	-7.80	-0.382	-7.449	-7.80	-0.351
cgcgcg~cgcgcg	-9.276	-9.12	0.156	-9.304	-9.12	0.184
cggccg~cggccg	-10.095	-9.90	0.195	-10.125	-9.90	0.225
cguacg~cguacg	-5.767	-5.35	0.417	-5.793	-5.35	0.443
cugcag~cugcag	-7.121	-7.11	0.011	-7.142	-7.11	0.032
gacguc~gacguc	-6.980	-7.35	-0.370	-7.007	-7.35	-0.343
gagaga~ucucuc	-6.776	-6.95	-0.174	-6.799	-6.95	-0.151
gagcuc~gagcuc	-7.811	-7.98	-0.169	-7.841	-7.98	-0.139
gagcug~cagcuc	-7.937	-7.49	0.447	-7.954	-7.49	0.464
gaggag~cuccuc	-8.059	-8.5	-0.441	-8.082	-8.50	-0.418
gcaacg~cguugc	-6.903	-7.01	-0.107	-6.937	-7.01	-0.073
gcaucg~cgaugc	-7.235	-7.26	-0.025	-7.264	-7.26	0.004

gcaugc~gcaugc	-7.685	-7.38	0.305	-7.716	-7.38	0.336
gccgcg~cgcggc	-10.788	-10.88	-0.092	-10.809	-10.88	-0.071
gccggc~gccggc	-11.361	-11.2	0.161	-11.389	-11.20	0.189
gcgccg~cggcgc	-10.788	-10.91	-0.122	-10.809	-10.91	-0.101
gcgcgc~gcgcgc	-10.541	-10.62	-0.079	-10.568	-10.62	-0.052
gcgcgg~ccgcgc	-10.788	-11.38	-0.592	-10.809	-11.38	-0.571
gcggcg~cgccgc	-10.788	-10.4	0.388	-10.809	-10.40	0.409
gcgucg~cgacgc	-8.598	-8.76	-0.162	-8.617	-8.76	-0.143
gcuacg~cguagc	-7.286	-7.56	-0.274	-7.305	-7.56	-0.255
gcuagc~gcuagc	-7.864	-7.92	-0.056	-7.893	-7.92	-0.027
gcugag~cucagc	-7.937	-7.72	0.217	-7.954	-7.72	0.234
ggaucc~ggaucc	-7.929	-7.44	0.489	-7.972	-7.44	0.532
ggcgcc~ggcgcc	-11.361	-11.33	0.031	-11.389	-11.33	0.059
ggcgcg~cgcgcc	-10.788	-10.78	0.008	-10.809	-10.78	0.029
gguacc~gguacc	-7.852	-7.35	0.502	-7.878	-7.35	0.528
gucgac~gucgac	-6.980	-7.09	-0.110	-7.007	-7.09	-0.083
gugcac~gugcac	-7.556	-7.65	-0.094	-7.571	-7.65	-0.079
gugcag~cugcac	-7.809	-7.67	0.139	-7.819	-7.67	0.149
guggug~caccac	-7.803	-7.67	0.133	-7.812	-7.67	0.142
gugucg~cgacac	-7.105	-7.18	-0.075	-7.119	-7.18	-0.061
ucauga~ucauga	-4.674	-4.31	0.364	-4.707	-4.31	0.397
uccgga~uccgga	-8.349	-7.79	0.559	-8.380	-7.79	0.590
ucgcga~ucgcga	-7.530	-6.85	0.680	-7.559	-6.85	0.709
ucuaga~ucuaga	-4.852	-4.95	-0.098	-4.884	-4.95	-0.066
ugauca~ugauca	-4.674	-5.05	-0.376	-4.707	-5.05	-0.343
ugcgca~ugcgca	-8.105	-8.22	-0.115	-8.124	-8.22	-0.096
uggcca~uggcca	-8.925	-8.99	-0.065	-8.945	-8.99	-0.045
aaggagg~ccuccuu	-9.512	-9.54	-0.028	-9.539	-9.54	-0.001
acuguca~ugacagu	-8.183	-7.92	0.263	-8.176	-7.92	0.256
agucuga~ucagacu	-8.311	-7.52	0.791	-8.311	-7.52	0.791
gacucag~cugaguc	-9.077	-9.05	0.027	-9.077	-9.05	0.027
gagugag~cucacuc	-9.077	-9.71	-0.633	-9.077	-9.71	-0.633
gucacug~cagugac	-8.949	-8.62	0.329	-8.942	-8.62	0.322
gcaacga~ucguugc	-8.950	-9.20	-0.250	-8.969	-9.20	-0.231
uaaggua~uaccuua	-6.306	-6.95	-0.644	-6.331	-6.95	-0.619
aacuaguu~aacuaguu	-6.391	-7.16	-0.769	-6.399	-7.16	-0.761
aaugcauu~aaugcauu	-6.341	-7.18	-0.839	-6.367	-7.18	-0.813
accuuugc~gcaaaggu	-10.545	-10.64	-0.095	-10.541	-10.64	-0.099
acuauagu~acuauagu	-6.978	-6.98	-0.002	-6.958	-6.98	-0.022
acuuaagu~acuuaagu	-6.391	-6.16	0.231	-6.399	-6.16	0.239

agagagag~cucucucu	-10.867	-11.12	-0.253	-10.832	-11.12	-0.288
agauaucu~agauaucu	-7.055	-6.58	0.475	-7.052	-6.58	0.472
aguauacu~aguauacu	-6.978	-6.80	0.178	-6.958	-6.80	0.158
aguuaacu~aguuaacu	-6.391	-6.36	0.031	-6.399	-6.36	0.039
auacguau~auacguau	-6.276	-6.53	-0.254	-6.268	-6.53	-0.262
aucuagau~aucuagau	-7.055	-7.20	-0.145	-7.052	-7.20	-0.148
augcgcau~augcgcau	-10.308	-10.17	0.138	-10.292	-10.17	0.122
auguacau~auguacau	-6.799	-6.49	0.309	-6.782	-6.49	0.292
caaaaaac~guuuuuug	-4.810	-4.61	0.200	-4.868	-4.61	0.258
caugcaug~caugcaug	-9.404	-9.67	-0.266	-9.381	-9.67	-0.289
cgacgcag~cugcgucg	-12.729	-12.32	0.409	-12.686	-12.32	0.366
cucgcaca~ugugcgag	-12.144	-12.11	0.034	-12.096	-12.11	-0.014
gaacguuc~gaacguuc	-8.855	-9.30	-0.445	-8.863	-9.30	-0.437
gagagaga~ucucucuc	-11.252	-11.80	-0.548	-11.217	-11.8	-0.583
gagaucuc~gagaucuc	-10.221	-10.11	0.111	-10.206	-10.11	0.096
gauauauc~gauauauc	-6.192	-6.09	0.102	-6.201	-6.09	0.111
gaugcauc~gaugcauc	-10.095	-10.12	-0.025	-10.080	-10.12	-0.040
gccauggc~gccauggc	-14.346	-15.06	-0.714	-14.318	-15.06	-0.742
gcugcgac~gucgcagc	-13.995	-13.93	0.065	-13.950	-13.93	0.020
ggcuucaa~uugaagcc	-10.713	-10.2	0.513	-10.711	-10.20	0.511
guauauac~guauauac	-6.115	-5.94	0.175	-6.108	-5.94	0.168
gucuagac~gucuagac	-10.144	-10.11	0.034	-10.112	-10.11	0.002
gugaucac~gugaucac	-9.965	-9.49	0.475	-9.936	-9.49	0.446
guucgaac~guucgaac	-8.855	-8.76	0.095	-8.863	-8.76	0.103
uagaucua~uagaucua	-7.314	-7.25	0.064	-7.299	-7.25	0.049
uaugcaua~uaugcaua	-7.188	-7.27	-0.082	-7.174	-7.27	-0.096
uccgcgca~ugcgcgga	-14.539	-14.59	-0.051	-14.496	-14.59	-0.094
uccuugca~ugcaagga	-11.478	-11.09	0.388	-11.452	-11.09	0.362
ucuauaga~ucuauaga	-7.314	-6.96	0.354	-7.299	-6.96	0.339
ugaccuca~ugagguca	-11.681	-12.34	-0.659	-11.634	-12.34	-0.706
uuccggaa~uuccggaa	-10.224	-10.79	-0.566	-10.236	-10.79	-0.554
uugcgcaa~uugcgcaa	-9.98	-10.18	-0.200	-9.980	-10.18	-0.200
uuggccaa~uuggccaa	-10.799	-11.00	-0.201	-10.801	-11.00	-0.199
uuguacaa~uuguacaa	-6.471	-6.70	-0.229	-6.470	-6.70	-0.230
caaaaaaag~cuuuuuuug	-5.531	-5.47	0.061	-5.568	-5.47	0.098
aagguuggaa~uuccaaccuu	-13.307	-12.69	0.617	-13.239	-12.69	0.549
aaaaaaauuuuuuu~aaaaaaauuuuuuu	-6.888	-6.69	0.198	-6.843	-6.69	0.153
rank:	13			16		
chiSq:	1.774			1.81		
residual:	0.059			0.06		

Table S2. Benchmark comparisons of the Turner energy rules [1] with the original and new free energy prediction methods for the standard benchmarks plus 4 additional 17 base pair sequences. The free energy data is adjusted for 150 mM salt the Owczarzy model for change of salt [5].

sequence	original method			new method		
	fitted weight (fit) [kcal/mol]	experimental Tm data (exp) [kcal/mol]	difference (exp-fit) [kcal/mol]	fitted weight (fit) [kcal/mol]	experimental Tm data (exp) [kcal/mol]	difference (exp-fit) [kcal/mol]
ccgg~ccgg	-3.989	-3.80	0.189	-2.890	-3.80	-0.910
cgcg~cgcg	-3.369	-2.91	0.459	-2.277	-2.91	-0.633
gcgc~gcgc	-5.009	-3.86	1.149	-3.583	-3.86	-0.277
ggcc~ggcc	-5.628	-4.62	1.008	-4.196	-4.62	-0.424
acgca~ugcgu	-4.883	-3.97	0.913	-4.212	-3.97	0.242
agcga~ucgcu	-5.093	-4.05	1.043	-4.400	-4.05	0.350
cacag~cugug	-3.795	-3.70	0.095	-3.200	-3.70	-0.500
cacug~cagug	-3.795	-2.34	1.455	-3.200	-2.34	0.860
gcacg~cgugc	-5.532	-5.30	0.232	-4.996	-5.30	-0.304
gcucg~cgagc	-5.742	-5.27	0.472	-5.184	-5.27	-0.086
accggu~accggu	-6.909	-7.55	-0.641	-7.065	-7.55	-0.485
agagag~cucucu	-5.676	-5.75	-0.074	-5.589	-5.75	-0.161
agcgcu~agcgcu	-7.311	-7.03	0.281	-7.265	-7.03	0.235
aggccu~aggccu	-7.930	-7.40	0.530	-7.878	-7.40	0.478
augcau~augcau	-3.861	-3.57	0.291	-3.776	-3.57	0.206
cacgug~cacgug	-5.272	-5.63	-0.358	-5.464	-5.63	-0.166
cagcug~cagcug	-6.294	-5.72	0.574	-6.277	-5.72	0.557
caugcg~cgcaug	-5.877	-6.04	-0.163	-6.073	-6.04	0.033
ccaugg~ccaugg	-6.106	-6.34	-0.234	-6.288	-6.34	-0.052
ccgcgg~ccgcgg	-8.748	-9.09	-0.342	-9.175	-9.09	0.085
ccuagg~ccuagg	-6.335	-6.84	-0.505	-6.408	-6.84	-0.432
cgcgcg~cgcgcg	-8.129	-8.37	-0.241	-8.562	-8.37	0.192
cggccg~cggccg	-8.748	-9.15	-0.402	-9.175	-9.15	0.025
cguacg~cguacg	-4.694	-4.39	0.304	-4.982	-4.39	0.592
cugcag~cugcag	-6.294	-6.15	0.144	-6.277	-6.15	0.127
gacguc~gacguc	-6.312	-6.39	-0.078	-6.333	-6.39	-0.057
gagaga~ucucuc	-6.266	-5.89	0.376	-6.047	-5.89	0.157
gagcuc~gagcuc	-7.333	-7.02	0.313	-7.146	-7.02	0.126
gagcug~cagcuc	-7.204	-6.53	0.674	-7.109	-6.53	0.579
gaggag~cuccuc	-7.214	-7.54	-0.326	-7.197	-7.54	-0.343

gcaacg~cguugc	-6.239	-6.05	0.189	-6.325	-6.05	0.275
gcaucg~cgaugc	-6.397	-6.30	0.097	-6.507	-6.30	0.207
gcaugc~gcaugc	-7.125	-6.42	0.705	-6.981	-6.42	0.561
gccgcg~cgcggc	-9.649	-10.13	-0.481	-9.919	-10.13	-0.211
gccggc~gccggc	-10.388	-10.45	-0.062	-10.481	-10.45	0.031
gcgccg~cggcgc	-9.649	-10.16	-0.511	-9.919	-10.16	-0.241
gcgcgc~gcgcgc	-9.768	-9.87	-0.102	-9.868	-9.87	-0.002
gcgcgg~ccgcgc	-9.649	-10.63	-0.981	-9.919	-10.63	-0.711
gcggcg~cgccgc	-9.649	-9.65	-0.001	-9.919	-9.65	0.269
gcgucg~cgacgc	-7.611	-7.91	-0.299	-7.845	-7.91	-0.065
gcuacg~cguagc	-6.415	-6.60	-0.185	-6.439	-6.60	-0.161
gcuagc~gcuagc	-7.354	-6.96	0.394	-7.101	-6.96	0.141
gcugag~cucagc	-7.204	-6.76	0.444	-7.109	-6.76	0.349
ggaucc~ggaucc	-7.145	-6.48	0.665	-7.157	-6.48	0.677
ggcgcc~ggcgcc	-10.388	-10.58	-0.192	-10.481	-10.58	-0.099
ggcgcg~cgcgcc	-9.649	-10.03	-0.381	-9.919	-10.03	-0.111
gguacc~gguacc	-6.953	-6.39	0.563	-6.901	-6.39	0.511
gucgac~gucgac	-6.312	-6.13	0.182	-6.333	-6.13	0.203
gugcac~gugcac	-6.912	-6.69	0.222	-6.770	-6.69	0.080
gugcag~cugcac	-6.994	-6.71	0.284	-6.921	-6.71	0.211
guggug~caccac	-6.793	-6.71	0.083	-6.821	-6.71	0.111
gugucg~cgacac	-6.183	-6.22	-0.037	-6.296	-6.22	0.076
ucauga~ucauga	-4.210	-3.15	1.060	-3.988	-3.15	0.838
uccgga~uccgga	-7.472	-6.83	0.642	-7.488	-6.83	0.658
ucgcga~ucgcga	-6.853	-5.89	0.963	-6.875	-5.89	0.985
ucuaga~ucuaga	-4.439	-3.79	0.649	-4.108	-3.79	0.318
ugauca~ugauca	-4.210	-3.89	0.320	-3.988	-3.89	0.098
ugcgca~ugcgca	-7.453	-7.26	0.193	-7.312	-7.26	0.052
uggcca~uggcca	-8.072	-8.03	0.042	-7.925	-8.03	-0.105
aaggagg~ccuccuu	-8.221	-8.52	-0.299	-8.551	-8.52	0.031
acuguca~ugacagu	-6.996	-6.82	0.176	-7.096	-6.82	0.276
agucuga~ucagacu	-7.207	-6.42	0.787	-7.284	-6.42	0.864
gacucag~cugaguc	-7.856	-8.03	-0.174	-8.068	-8.03	0.038
gagugag~cucacuc	-7.856	-8.69	-0.834	-8.068	-8.69	-0.622
gucacug~cagugac	-7.645	-7.60	0.045	-7.880	-7.60	0.280
gcaacga~ucguugc	-7.981	-8.18	-0.199	-8.208	-8.18	0.028
uaaggua~uaccuua	-5.359	-5.76	-0.401	-5.348	-5.76	-0.412
aacuaguu~aacuaguu	-5.292	-5.95	-0.658	-5.418	-5.95	-0.532
aaugcauu~aaugcauu	-5.276	-5.97	-0.694	-5.509	-5.97	-0.461
accuuugc~gcaaaggu	-9.228	-9.58	-0.352	-9.455	-9.58	-0.125

acuauagu~acuauagu	-5.414	-5.77	-0.356	-5.526	-5.77	-0.244
acuuaagu~acuuaagu	-5.292	-4.95	0.342	-5.418	-4.95	0.468
agagagag~cucucucu	-9.218	-10.06	-0.842	-9.438	-10.06	-0.622
agauaucu~agauaucu	-5.607	-5.37	0.237	-5.782	-5.37	0.412
aguauacu~aguauacu	-5.414	-5.59	-0.176	-5.526	-5.59	-0.064
aguuaacu~aguuaacu	-5.292	-5.15	0.142	-5.418	-5.15	0.268
auacguau~auacguau	-4.607	-5.32	-0.713	-4.924	-5.32	-0.396
aucuagau~aucuagau	-5.607	-5.99	-0.383	-5.782	-5.99	-0.208
augcgcau~augcgcau	-8.62	-9.11	-0.490	-8.986	-9.11	-0.124
auguacau~auguacau	-5.185	-5.28	-0.095	-5.406	-5.28	0.126
caaaaaac~guuuuuug	-4.311	-3.40	0.911	-4.581	-3.40	1.181
caugcaug~caugcaug	-7.603	-8.61	-1.007	-7.998	-8.61	-0.612
cgacgcag~cugcgucg	-10.634	-11.41	-0.776	-11.259	-11.41	-0.151
cucgcaca~ugugcgag	-10.295	-11.13	-0.835	-10.634	-11.13	-0.496
gaacguuc~gaacguuc	-7.727	-8.24	-0.513	-8.066	-8.24	-0.174
gagagaga~ucucucuc	-9.808	-10.74	-0.932	-9.896	-10.74	-0.844
gagaucuc~gagaucuc	-8.850	-9.05	-0.200	-9.032	-9.05	-0.018
gauauauc~gauauauc	-4.843	-4.88	-0.037	-5.016	-4.88	0.136
gaugcauc~gaugcauc	-8.642	-9.06	-0.418	-8.867	-9.06	-0.193
gccauggc~gccauggc	-12.504	-14.15	-1.646	-12.804	-14.15	-1.346
gcugcgac~gucgcagc	-12.273	-13.02	-0.747	-12.566	-13.02	-0.454
ggcuucaa~uugaagcc	-9.509	-9.14	0.369	-9.666	-9.14	0.526
guauauac~guauauac	-4.650	-4.73	-0.080	-4.760	-4.73	0.030
gucuagac~gucuagac	-8.657	-9.05	-0.393	-8.776	-9.05	-0.274
gugaucac~gugaucac	-8.428	-8.43	-0.002	-8.656	-8.43	0.226
guucgaac~guucgaac	-7.727	-7.70	0.027	-8.066	-7.70	0.366
uagaucua~uagaucua	-5.977	-6.04	-0.063	-5.948	-6.04	-0.092
uaugcaua~uaugcaua	-5.770	-6.06	-0.290	-5.783	-6.06	-0.277
uccgcgca~ugcgcgga	-12.613	-13.68	-1.067	-13.007	-13.68	-0.673
uccuugca~ugcaagga	-10.023	-10.03	-0.007	-10.126	-10.03	0.096
ucuauaga~ucuauaga	-5.977	-5.75	0.227	-5.948	-5.75	0.198
ugaccuca~ugagguca	-9.967	-11.28	-1.313	-10.097	-11.28	-1.183
uuccggaa~uuccggaa	-8.888	-9.73	-0.842	-9.220	-9.73	-0.510
uugcgcaa~uugcgcaa	-8.868	-9.12	-0.252	-9.045	-9.12	-0.075
uuggccaa~uuggccaa	-9.488	-9.94	-0.452	-9.658	-9.94	-0.282
uuguacaa~uuguacaa	-5.433	-5.49	-0.057	-5.465	-5.49	-0.025
caaaaaaag~cuuuuuuug	-4.710	-4.24	0.470	-4.985	-4.24	0.745
aagguuggaa~uuccaaccuu	-11.167	-11.57	-0.403	-11.250	-11.57	-0.320
aaaaaaauuuuuuu~aaaaaaauuuuuuu	-5.478	-5.32	0.158	-4.867	-5.32	-0.453
AUGAUCGUUUCGUACUG~][CAGUACGAAACGAUCAU	-19.896	-20.63	-0.734	-19.187	-20.63	-1.443

AUGAUCGGGGCGUACUG~ CAGUACGCCCCGAUCAU	-25.897	-23.07	-2.827	-25.350	-23.07	2.280
AUGAUCGGCGCGUACUG~ CAGUACGCGCCGAUCAU	-25.277	-23.63	-1.647	-24.737	-23.63	1.107
AUGAUCGACACGUACUG~ CAGUACGUGUCGAUCAU	-21.811	-22.05	-0.239	-21.114	-22.05	-0.936
rank:	13			16		
chisq:	6.179			4.019		
residual:	0.198			0.134		

Table S3. An example of the results of an individual in the final 102nd generation; the 8179th calculation. The scoring parameters are explained in Section 2.6 of the main text and Equations (9)-(14). At the top, titled “SymRecords”, 8179 is the iteration number, the next 6 numbers are the parameters shown in Tables 1 and 2 for the New Model, the next 10 parameters are the 2-nt bp parameters in the same order as Tables 1 and 2, the next 4 parameters are values from the scoring function, and the last number is the score. The next heading, “tested_structures:” are the specific scores found for each of the fitted duplexes. The first column is the sequence name, followed by $(n_{i,bp}^{ref} - n_{i,match}^{pred})^2$, $(s_{i,stem}^{ref} - s_{i,match}^{pred})^2$, $(V_i^{ref} - V_i^{pred})^2$, and $(E_{i,xpt}^{ref} - E_{i,calc}^{pred})^2$, which correspond to Equations (9) through (12). The next heading, “weights”, specifies the tally of the scores. Following the column “wt name”, in the column “weight” wts_dBP is w_{bp}, wts_dstem is w_{stem}, wts_dErel is w_{sc} and wts_dExep is w_{xpt}. Under the column <rmsd^2>, the two values are the variances δS_{bp} and δS_{stem}. Under <dE^2>, the two values are the variances δV_{sc} and δE_{xpt}. Finally, at the bottom, the score is also indicated again. Note that this particular sample is *provided as an example of the output from the GA* and only reflects a particular calculation (in this case the last one), not a final result as such.

```
SymRecords:
8179        0.32      0.81      1.18      1.78      1.08      3.11     -1.42
-2.49     -2.40     -1.31     -2.54     -3.61     -2.80     -2.83     -3.75     -
1.71 |   0.0000    0.0000    0.0000    0.0008  |     99.8332
tested_structures:
1                    0       0        0.00        0.15
2                    0       0        0.00        0.37
3                    0       0       -0.00        0.37
4                    0       0        0.00        0.28
5                    0       0       -0.00        0.08
6                    0       0       -0.00        0.20
7                    0       0        0.00       -0.45
y_1                  0       0        0.00        0.91
8                    0       0        0.00       -0.31
9                    0       0        0.00       -0.08
10                   0       0        0.00       -0.27
y_2                  0       0       -0.00        0.10
11                   0       0        0.00        0.35
12                   0       0        0.00        0.65
y_3                  0       0       -0.00       -0.04
13                   0       0        0.00        0.32
14                   0       0        0.00        1.00
x_1                  0       0       -0.00       -0.01
15                   0       0        0.00        0.36
16                   0       0       -0.00        0.78
17                   0       0        0.00       -0.02
18                   0       0        0.00        0.83
19                   0       0       -0.00        0.72
y_4                  0       0        0.00        0.99
20                   0       0       -0.00        0.57
21                   0       0       -0.00        0.14
22                   0       0       -0.00       -0.42
23                   0       0       -0.00        0.28
x_2                  0       0        0.00        0.48
24                   0       0       -0.00       -0.38
25                   0       0        0.00        0.04
26                   0       0        0.00        0.02
27                   0       0        0.00        0.56
```

28	0	0	-0.00	-0.12
29	0	0	0.00	0.37
30	0	0	-0.00	-0.15
31	0	0	-0.00	0.28
32	0	0	-0.00	-0.62
33	0	0	-0.00	0.36
34	0	0	-0.00	-0.04
35	0	0	0.00	-0.36
36	0	0	0.00	0.14
x_3	0	0	0.00	0.25
37	0	0	0.00	0.80
38	0	0	0.00	0.24
39	0	0	-0.00	-0.02
40	0	0	0.00	0.61
41	0	0	-0.00	0.40
42	0	0	0.00	0.21
x_4	0	0	-0.00	0.10
43	0	0	0.00	0.05
44	0	0	0.00	0.02
45	0	0	0.00	0.98
46	0	0	0.00	1.13
47	0	0	-0.00	1.40
48	0	0	0.00	0.46
49	0	0	-0.00	0.24
50	0	0	-0.00	0.40
y_5	0	0	-0.00	0.30
51	0	0	0.00	0.47
52	0	0	0.00	0.30
53	0	0	-0.00	0.90
54	0	0	0.00	0.18
55	0	0	0.00	-0.48
56	0	0	0.00	0.41
y_6	0	0	0.00	-0.44
x_5	0	0	0.00	-0.68
57	0	0	0.00	-0.31
58	0	0	-0.00	-0.37
59	0	0	0.00	0.32
60	0	0	0.00	0.05
61	0	0	0.00	0.69
62	0	0	0.00	0.30
63	0	0	-0.00	0.73
y_7	0	0	0.00	0.23
64	0	0	0.00	0.49
65	0	0	0.00	-0.19
66	0	0	-0.00	0.11
67	0	0	0.00	0.35
68	0	0	0.00	0.42
69	0	0	0.00	0.85
70	0	0	-0.00	0.19
71	0	0	0.00	0.62
72	0	0	0.00	-0.24
73	0	0	-0.00	0.31
y_8	0	0	-0.00	-0.76
x_6	0	0	0.00	0.65
74	0	0	-0.00	0.25
75	0	0	0.00	0.32
x_7	0	0	0.00	-0.62
x_8	0	0	0.00	-0.04
76	0	0	0.00	0.29

77	0	0	0.00	0.12
78	0	0	0.00	0.37
y_9	0	0	0.00	0.87
79	0	0	-0.00	0.85
80	0	0	0.00	0.46
81	0	0	0.00	0.12
x_9	0	0	-0.00	0.01
82	0	0	-0.00	0.52
83	0	0	0.00	0.75
84	0	0	0.00	-0.58
85	0	0	-0.00	0.25
86	0	0	0.00	0.56
87	0	0	0.00	0.41
88	0	0	0.00	0.43
89	0	0	-0.00	0.88
90	0	0	0.00	0.65
y10	0	0	0.00	1.28

weights:

wt name	weight	<rmsd^2>	<dE^2>
wts_dBP	2.0000	0.0000	
wts_dstem	1.0000	0.0000	
wts_dErel	1.0000		0.0000
wts_dEexp	2.0000		0.0008
totals:		0.0000	0.0017

score(100*exp[-(...)]): 99.83324435

// --------- end record 8179

References

1. Xia, T.; SantaLucia, Jr.,J.; Burkard, M.E.; Kierzek, R.; Schroeder, S.J.; Jiao, X.; Cox, C.; Turner, D.H. Thermodynamic parameters for an expanded nearest-neighbor model for formation of RNA duplexes with Watson–Crick base pairs. *Biochemistry* **1988**, *37*, 14719–14735.
2. Gray, D.M.; Tinoco, I. A new approach to the study of sequence-dependent properties of polynucleotides. *Biopolymers* **1970**, *9*, 223–244.
3. Borer, P.N.; Dengler, B.; Tinoco Jr., I.; Uhlenbeck, O.C. Stability of ribonucleic acid double-stranded helices. *J. Mol. Biol.* **1974**, *86*, 843–853.
4. Applequist, J.; Damle, V. Thermodynamics of the helix-coil equilibrium in oligoadenylic acid from hypochromicity studies. *J. Am. Chem. Soc.* **1965**, *87*, 1450–1458.
5. Chen, Z.; Znosko, B.M. Effect of sodium ions on RNA duplex stability. *Biochemistry* **2013**, *52*, 7477–7485.
